# Data of compressible multi-material flow simulations utilizing an efficient bimaterial Riemann problem solver

**DOI:** 10.1016/j.dib.2024.110081

**Published:** 2024-01-23

**Authors:** Wentao Ma, Xuning Zhao, Shafquat Islam, Aditya Narkhede, Kevin Wang

**Affiliations:** Department of Aerospace and Ocean Engineering, Virginia Tech, Blacksburg, VA 24061, USA

**Keywords:** Multiphase flow, Multi-material flow, Riemann problem, Equation of state, Compressible flow

## Abstract

This paper presents fluid dynamics simulation data associated with two test cases in the related research article [1]. In this article, an efficient bimaterial Riemann problem solver is proposed to accelerate multi-material flow simulations that involve complex thermodynamic equations of state and strong discontinuities across material interfaces. The first test case is a one-dimensional benchmark problem, featuring large density jump (4 orders of magnitude) and drastically different thermodynamics relations across a material interface. The second test case simulates the nucleation of a pear-shaped vapor bubble induced by long-pulsed laser in water. This multiphysics simulation combines laser radiation, phase transition (vaporization), non-spherical bubble expansion, and the emission of acoustic and shock waves. Both test cases are performed using the M2C solver, which solves the three-dimensional Eulerian Navier-Stokes equations, utilizing the accelerated bimaterial Riemann solver. Source codes provided in this paper include the M2C solver and a standalone version of the accelerated Riemann problem solver. These source codes serve as references for researchers seeking to implement the acceleration algorithms introduced in the related research article. Simulation data provided include fluid pressure, velocity, density, laser radiance and bubble dynamics. The input files and the workflow to perform the simulations are also provided. These files, together with the source codes, allow researchers to replicate the simulation results presented in the research article, which can be a starting point for new research in laser-induced cavitation, bubble dynamics, and multiphase flow in general.

Specifications Table

**Table:** 

Subject	Engineering/Computational Mechanics
Specific subject area	Compressible multiphase flow, Computational fluid dynamics
Data format	Raw Visualized
Type of data	Source code Image Video ASCII files (simulation inputs and outputs)
Data collection	The simulation output data was generated using the M2C solver and the Tinkercliffs computer cluster at Virginia Tech.
Data source location	• Institution: Virginia Tech • City/Town/Region: Blacksburg, VA • Country: USA
Data accessibility	Repository name: Mendeley Data Data identification number: 10.17632/b7×55v2knk.3 [2] Direct URL to data: https://data.mendeley.com/datasets/b7x55v2knk/3
Related research article	[1] W. Ma, X. Zhao, S. Islam, A. Narkhede, K. Wang, Efficient solution of bimaterial Riemann problems for compressible multi-material flow simulations, Journal of Computational Physics, 493 (2023) 112,474. https://doi.org/10.1016/j.jcp.2023.112474

## 1 Value of the Data

• The data set in this paper come from two test cases in the related research article [1], which develops an efficient bimaterial Riemann problem solver that significantly accelerates multi-material flow simulations featuring arbitrary complex equations of state (EOS) and strong discontinuity across material interfaces. The associated source codes are provided to give researchers references to implement the acceleration algorithms introduced in the related research article.
• The first test case is a one-dimensional benchmark problem, with a large density jump (4 orders of magnitude) and drastically different EOS across the material interface. This simulation provides a challenging test case for researchers who implement the acceleration algorithms in the related research article [1], or who develop their own multi-material flow solution algorithms.
• The second test case is about the nucleation and expansion of a pear-shaped bubble induced by a long-pulse laser. In laboratory experiments using the same type of laser, the same shape has been observed [3]. This simulation considers various realistic physical phenomena, including laser radiation, vaporization, non-spherical bubble expansion, and the emission of acoustic and shock waves.
• With the data and information provided in this paper, researchers can replicate the two simulations and use them as a starting point to study related problems involving bubble dynamics, laser-fluid interaction, vaporization, and multiphase flow.

## 2 Background

In compressible multi-material flow simulations, an unresolved challenge lies in computing advective fluxes across material interfaces that separate substantially different thermodynamic states and relations. A popular approach involves the local construction of Riemann problems and utilizing their exact solutions for flux computation. However, for complex equations of state, obtaining the exact solution of a Riemann problem proves computationally expensive due to the nested loops required. Multiplied by the large number of Riemann problems generated throughout a simulation, the resulting computational expenses are often prohibitive. In response to this challenge, the related research article [1] introduces a new Riemann problem solver designed to accelerate the solution of bimaterial Riemann problems without resorting to approximations or offline precomputation tasks. Consequently, the acceleration achieved by this new solver significantly enhances the performance of the advective flux calculator—a critical component akin to the engine of multi-material flow› solvers. Noteworthy speed gains, ranging from 18 to 81 times, were observed in various test cases, including underwater explosion, laser-induced cavitation, and hypervelocity impact. This data paper offers selected input and results files linked to two test cases in the original research article [1]. Complete source codes are also made available. The resource introduced here not only facilitates reproduction of the simulation results but also serves as a starting point for new research in bubble dynamics, vaporization, and multiphase flow in general.

## 3 Data Description

**Fig. 1:**
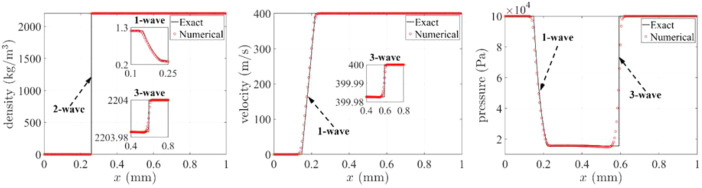
1D benchmark problem: Density, velocity, and pressure distributions at t=0.15μs (adapted from Figure 10 in the related research article [1]).

The data set in this paper is associated with two test cases in the related research article [1], which significantly accelerates challenging multi-material flow simulations by developing a new, efficient bimaterial Riemann problem solver. Test 1 in this data paper is a one-dimensional (1D) benchmark problem, which models a condensed phase (soda lime glass) moving away from a gas (air) at a high speed (400 m/s). The density, velocity, and pressure distributions at t=0.15μs are plotted in Fig. 1. At any time t>0, the density ratio across the material interface reaches 4 orders of magnitude, from 0.3 kg/m^3^ to 2203.98 kg/m^3^, which challenges the robustness of multi-material flow solvers. The simulation result data are generated using the high-fidelity multiphase computational fluid dynamics solver M2C [4], which utilizes the accelerated bimaterial Riemann solver at material interfaces. The exact solution is generated using a standalone version of the efficient bimaterial Riemann problem solver [5]. The source codes of these two solvers are provided in the online data repository [2] (see Table 1). The file paths are relative to the main directory, i.e., the ***EfficientRiemann_DataSet*** folder in the online repository.

**Table 1:** Source codes used to generate the results data.

File path	File description
SourceCodes/m2c	Multiphase CFD solver M2C [4]
SourceCodes/riemann	Standalone bimaterial Riemann problem solver [5]

In the online data repository, Test 1 files are listed in Table 2, including both the necessary input files for launching the simulation and selected simulation outputs. Again, the file paths are expressed relative to the main directory, i.e., the ***EfficientRiemann_DataSet*** folder in online repository. Specifically, the *Image.zip* file includes the subfigures in Fig. 1.

**Table 2:** Simulation input and output files of Test1, 1D benchmark problem.

File path	File description
1dBenchmark/Simulation/input.st	Input parameters for M2C solver
1dBenchmark/Simulation/tinkercliffs_sbatch.sh	The bash script for submitting the simulation on Tinkercliffs
1dBenchmark/Simulation/log.out	The screen outputs generated by M2C solver
1dBenchmark/Images.zip	Images generated using the simulation results
1dBenchmark/Simulation/input_standaloneRiemann.st	Input parameters for the standalone Riemann solver

**Fig. 2:**
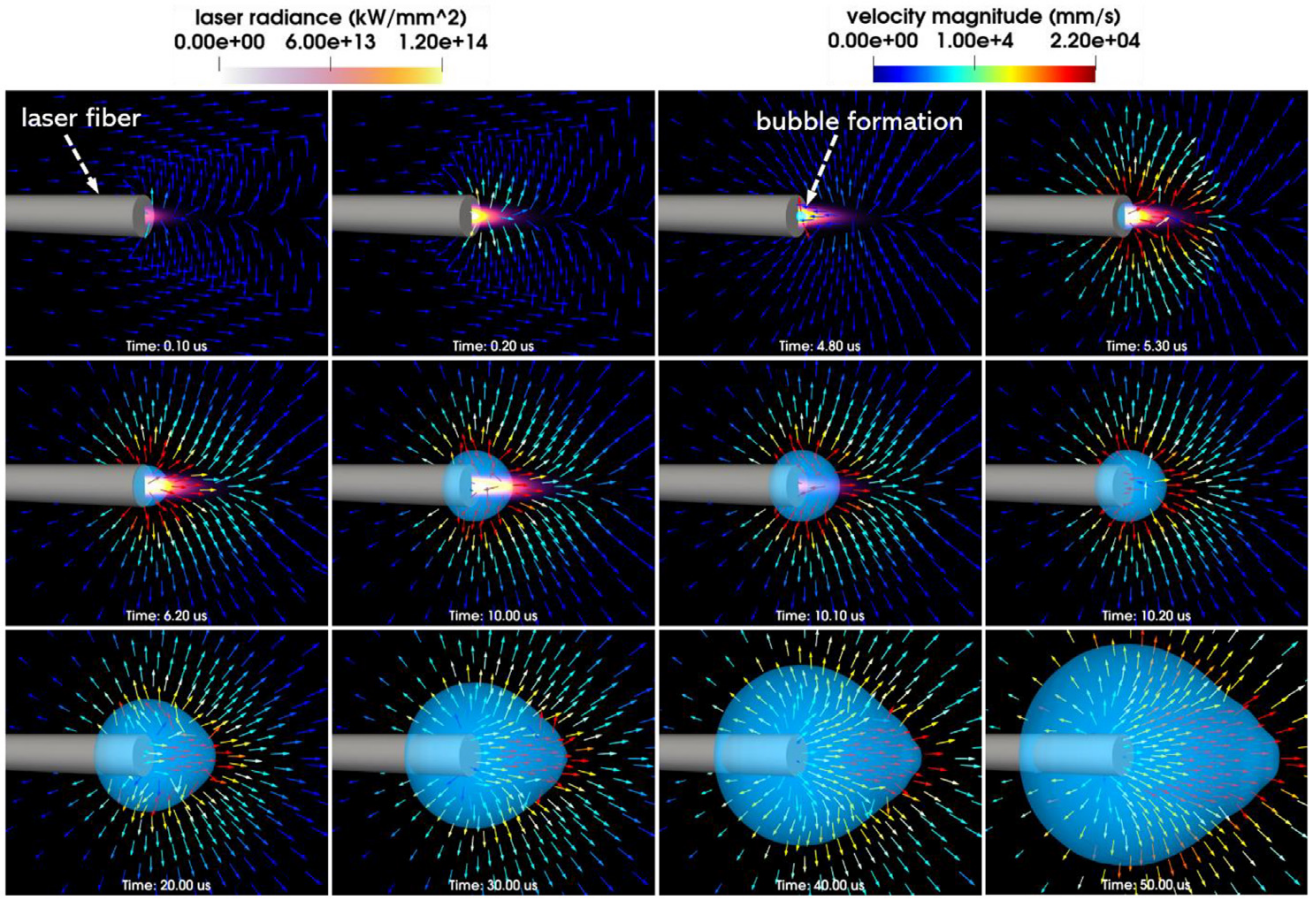
Laser-induced cavitation: Laser radiation, bubble formation, and the fluid velocity field.

**Fig. 3:**
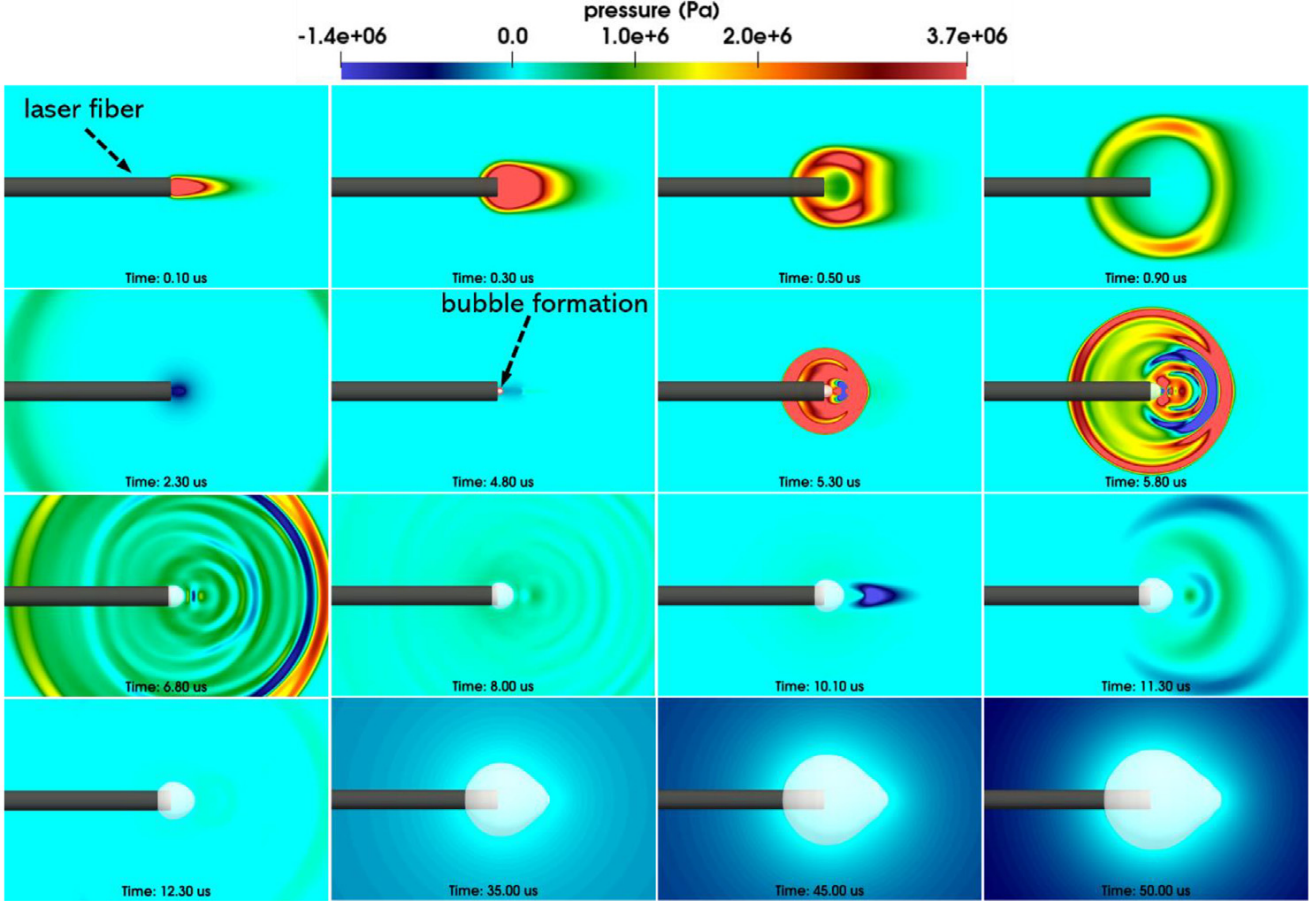
Laser-induced cavitation: The pressure field.

Test 2 in this data paper simulates a pear-shaped bubble induced by a long-pulse laser. The simulation generates a variety of output data, including but not limited to laser radiance, fluid pressure, velocity, temperature, and level-set information used for tracking liquid-gas interfaces. Fig. 2 showcases a series of images that depict the evolution of laser radiance fields and the formation of a non-spherical bubble. Each sub-figure corresponds to a specific time instant, as indicated at the bottom. In Fig. 3, a sequence of images illustrates the progression of pressure fields and the expansion of the pear-shaped bubble. More detailed numerical experiment setup, such as geometry of the laser radiation domain, spatial profile of laser radiance, and the temporal profile of laser power, can be found in [6].

Table 3 lists the files associated with Test 2 in the online data repository. Again, the file paths are expressed relative to the main directory. The files include input files that are necessary for launching the simulations and selected simulation outputs. In the file *Images.zip*, 1002 images are included to show the progression of the bubble dynamics, as well as the velocity,pressure, and laser radiance fields. Two animations created using these images are placed in the *Videos* folder.A file of mesh information is also outputted and placed in the *Simulation* folder.

**Table 3:** Simulation input and output files of Test2, laser-induced cavitation.

File path	File description
LaserBubble/Simulation/input.st	Input parameters
LaserBubble/Simulation/Laser_power.txt	The temporal profile of laser power (input)
LaserBubble/Simulation/tinkercliffs_sbatch.sh	The bash script for submitting the simulation on Tinkercliffs
LaserBubble/Simulation/log.out	The screen outputs generated by the simulation
LaserBubble/ Simulation/meshinfo.txt	Mesh information (output)
LaserBubble/Images.zip	Images generated using the simulation results
LaserBubble/Videos/radiance_velocity.avi	An animation of the laser radiance and the velocity fields
LaserBubble/Videos/pressure.avi	An animation of the pressure filed

## 4 Experimental Design, Materials and Methods

### 4.1 Test1: 1D benchmark problem

The simulation domain of this test case extends from x=0 to 1.0 mm. The material interface is initially at x=0.2 mm. The initial condition is

$$\left(\rho ,u,p,\text{EOS}\right)=\left\{ \begin{array}{c} \left(1.2\times 10^{-6}\;g/mm^{3},0.0\;\text{mm}/s,1.0\times 10^{5}\;\text{Pa},\;\text{stiffened}\;\text{gas}\;\right)\;\;\;\;\;\text{if}\;x<0.2\;\text{mm}\\ \left(2.204\times 10^{-3}\;g/mm^{3},4.0\times 10^{5}\;\text{mm}/s,1.0\times 10^{5}\;\text{Pa},\;\text{Mie}-\text{Gr}\ddot{u}\text{neisen}\;\right)\;\text{if}\;x>0.2\;\text{mm}. \end{array}\right.$$

The parameters in the stiffened gas EOS are given by γ=1.4, $e_{c}=0$, b=0, and $p_{c}=0$ [1]. Therefore, gas phase essentially degenerates to perfect gas EOS. The parameters in the Mie-Grüneisen EOS [1] are given by $\rho_{0}=2.204\times 10^{-3}$ g/mm^3^, $c_{0}=2.22\times 10^{6}$ mm/s, s=1.61, and $\Gamma_{0}=0.65.$

### 4.2 Test2: laser-induced cavitation

In Table 4, the characteristics of the laser are detailed. Table 5 outlines the properties of liquid water. Additionally, Table 6 provides the physical attributes of the water vapor confined within the bubble.

**Table 4:** Properties of laser beam [1].

Type	Wavelengh	Laser fiber radius	Beam waist	Divergence	Peak power
Holmium: YAG	2120 nm	0.1825 mm	0.12 mm	7.5°	2.854 kW

**Table 5:** Properties of liquid water [1].

Initial pressure	Initial density	Initial temperature	Initial velocity
100 kPa	0.001 g/mm^3^	273.15 K	0 mm/s

Specific heat at constant pressure	Laser absorption coefficient	Vaporization temperature	Latent heat

$4.\;2\;\times \;10^{9}$ mm^2^/(s^2^K)	2.42 mm^−1^	373.15 K	2256.4 J/g

**Table 6:** Properties of water vapor [1].

Specific heat at constant pressure	Laser absorption coefficient	Heat capacity
$2.0\;\times \;10^{9}\;$mm^2^/(s^2^K)	0.01 mm^−1^	1.34 [7]

The modeling of liquid water employs the stiffened gas equation of state (EOS) [7], characterized by γ=6.12 and $p_{c}=343$ MPa. These two parameters are determined as fitting parameters using shockwave Hugoniot data for water [8]. Meanwhile, the representation of water vapor is based on the perfect gas EOS.

### 4.3 Solver and external libraries

The multi-material flow simulations were performed using the M2C solver. The exact solution in Test 1 was generated by a standalone Riemann solver. These two solvers are uploaded to the online data repository, see Table 1. Readers can also access these two solvers on GitHub [4]. The versions of external libraries used by these two solvers are listed in Table 7.

**Table 7:** External libraries used by M2C.

Name	Version
Boost	1.71.0
Intel MPI	2018.5.288
Eigen	3.3.8
METIS	5.1.0
MUMPS	5.2.1

### 4.4 Simulation process

For every multi-material flow test case, the simulation parameters are defined within the file ***input.st***; Specifically for Test 2, the temporal profile of laser power is specified in ***laser_power.txt***. The simulations were launched on the Tinkercliffs computer cluster at Virginia Tech, using their respective sbatch script ***tinkercliffs_sbatch.sh***.

In Test 1, the simulation is carried out on a one-dimensional mesh with 200 elements. The time step size was around 0.95 ns. After 158 time steps (t=0.15μs), the simulation was terminated.

In Test 2, we conduct the simulation on a two-dimensional mesh, taking advantage of the problem's cylindrical symmetry. The fluid mesh contains around 338, 000 finite volume cells. Throughout the simulation, the computational domain is divided into 256 subdomains, with each one assigned to one CPU core. The time step size ranged between 0.2 and 0.5 ns. After completing 369,817 time steps, which is equivalent to t=50μs, the simulation was successfully terminated.

## Limitations

Not applicable

## Ethics Statement

The authors have read and follow the ethical requirements for publication in Data in Brief and confirm that the current work does not involve human subjects, animal experiments, or any data collected from social media platforms.

## CRediT authorship contribution statement

**Wentao Ma:** Conceptualization, Methodology, Software, Validation, Investigation, Writing – original draft, Writing – review & editing. **Xuning Zhao:** Methodology, Software, Validation. **Shafquat Islam:** Methodology, Software, Validation. **Aditya Narkhede:** Methodology, Software. **Kevin Wang:** Conceptualization, Methodology, Software, Investigation, Writing – original draft, Writing – review & editing, Funding acquisition.

## Data Availability

Data of compressible multi-material flow simulations utilizing an efficient bimaterial Riemann problem solver (Original data) (Mendeley Data)
